# The indirect relationship of self-kindness and anxiety and pain intensity through the affectivity: prospective study in Polish patients with chronic pain

**DOI:** 10.3389/fpain.2026.1864709

**Published:** 2026-10-30

**Authors:** Marcin Wnuk, Nicole Goldsztajn

**Affiliations:** 1 Department of Work and Organizational Psychology, Adam Mickiewicz University, Poznań, Poland; 2 Poznań University of Medical Sciences, Poznań, Poland

**Keywords:** anxiety, negative affect, patients with chronic pain, positive affect, self-kindness

## Abstract

**Introduction:**

As previous research suggests, self-compassion may be an effective way of managing pain and protecting against mental health disorders in patients struggling with chronic pain. On the other hand, the role of emotional well-being in these relationships remains unexplained. Based on the broaden-and-build theory of positive emotions, this study assumed that self-kindness can be indirectly related to pain intensity and anxiety via positive and negative affect.

**Methods:**

This prospective study was conducted at two time points, with a 4-month break between the first and second research waves, on the 58 participants struggling with chronic pain for at least three months despite pharmacological treatment.

**Results:**

Research revealed that self-kindness positively predicts positive affect and negatively predicts negative affect. Positive affect was not a statistically significant predictor of anxiety, but it statistically significantly predicted pain intensity. Negative affect was found to be a positive predictor of anxiety, but not intensity. The indirect negative relationship between self-kindness and pain intensity, through positive affect, was found to be the same as the negative relationship between self-kindness and anxiety via negative affect.

**Conclusion:**

Enhancing self-kindness as a strength of character, through increased positive affect and decreased negative affect, may be related to pain intensity and anxiety symptoms in patients with chronic pain. The need for using self-kindness techniques, both in daily life and as part of a strategic intervention for a group of patients with chronic pain, was discussed.

## Introduction

Chronic pain is a prevalent and complex condition shown to have been linked to psychological and social factors that greatly affect the quality of life of those afflicted (1). It is defined as pain that is constant or sporadic in nature, persisting for more than 3 months (2). In addition to the physical and mental burden that is placed on patients, chronic pain also places a significant financial and social strain on healthcare services (2).

Although there are many options for pharmacological management of chronic pain, the psychological approach promoting mental health and well-being is not extensively studied or routinely implemented. One promising psychological resource, which can be profitable in this group of patients, is self-compassion. Self-compassion is defined as an understanding or positive outlook on oneself when considering any flaws, shortcomings, and imperfections concurrently present (3). It is the ability to react with compassion when confronted with pain, failure or feelings of inadequacy (4). The idea of self-compassion consisting of 3 components was introduced by Neff and Dahm (3, which include self-kindness, mindfulness, and common humanity. Self-kindness encourages the avoidance of self-criticism in times of adversity, while mindfulness allows for the recognition of self-critical or emotionally painful thoughts and holding them in acknowledgement rather than fixating on them (4). Common humanity is relating one's experience to that of humanity as a whole as opposed to an individual event that can bring on feelings of isolation (4).

Self-compassion as a strength of character (5) and coping strategy (6), can be successfully developed by patients with chronic pain on their own as a psychological resource in the management of pain and dealing with mental health problems (4, 7–9). Compassion-Focused Therapy (10–12) and Mindful Self-Compassion (13) are two leading therapeutic approaches used to actively develop self-compassion, shifting it from an occasional state into a stable, enduring disposition or trait. Compassion-Focused Therapy (CFT) is a form of psychotherapy designed to help people who experience high levels of shame and self-criticism, integrating techniques from cognitive behavioral therapy (CBT) with insights from neuroscience, evolutionary psychology, and Buddhist mindfulness practices (10). Mindful Self-compassion is an empirical, evidence-based training program that combines the skills of mindfulness with the emotional practice of self-compassion to build emotional resilience based on three opposing dimensions: mindfulness versus over-identification, common humanity versus isolation, self-kindness versus self-judgment (13). In this study, self-kindness as an indicator of a self-compassionate approach was used in contrast to self-judgment as the opposite phenomenon to self-kindness. Self-kindness means treating yourself with the same warmth, empathy, and understanding as you would extend to a close friend.

Also, psychological interventions oriented to improve self-compassion resources yielded fruitful effects in patients suffering from chronic pain (1, 14–17), giving relief from pain (18, 19), and protection from mental health deterioration (19). Chapin et al. (16) show the effectiveness of compassion meditation intervention in patients with chronic pain in reduced pain severity, anger, and elevation of pain acceptance. Also, the mindful self-compassion program turned out to be effective in terms of strengthening pain acceptance, weakening pain interference, pain catastrophizing, and anxiety (20).

There are some relatively simple methods, which do not require special training to implement, like preparing self-compassionate letters, visualizing a compassionate self, or using self-compassion mantras (21). It seems especially important in patients with chronic pain, who struggling with pain need to confront the co-occurrence of poor mental health conditions (22, 23). Research reported that self-compassion is positively correlated with emotional resilience and positive affect, and negatively related to levels of depression, pain-related anxiety, and analgesic use (4). Guerra et al. (24) found that among palliative patients, self-compassion negatively predicted depression. Chang et al. (6) showed that isolation, as a one dimension of self-compassion, was related to pain severity in fibromyalgia patients. Similar conclusions emerge from prospective research indicating that self-compassion is an antecedent of pain and mental health indicators. For example, in one longitudinal study, stronger uncompassionate self-responding predicted more severe pain-related anxiety and pain catastrophizing (8). Also, in the Carvalho et al. (7) research, baseline self-compassion predicted fewer depressive symptoms at both the 6- and 12-month time points.

Notably, the psychological mechanism underlying the link between self-compassion and pain intensity and the mental health of patients with chronic pain remains unclear. A previous study suggests that, from a psychophysiological perspective, self-compassion is involved in regulating pain through heart rate variability and the secretion of oxytocin and endorphins (9). Although, there is a lack of studies that explain how psychological self-compassion can influence pain and mental health. In this study, we propose the positive and negative effects as mediators in the relationship between self-kindness and pain intensity, as well as self-kindness and anxiety as a mental health indicator. The premise for the test affectivity is caused by the fact that previous research suggests that emotion regulation may be a significant consequence of self-compassionate reactions (4, 8, 25, 26).

The theoretical basis for this prospective study is the broaden-and-build theory of positive emotions (27), which, through positive and negative affect, links self-compassion to positive outcomes. In line with this approach, the long-term personal physical, intellectual, social, and psychological resources required for well-being are affected by positive emotions that occur, such as joy, interest, and contentment, which broaden an individual's momentary mindset—expanding awareness and encouraging novel, creative thoughts and actions (28). This repertoire of positive emotions can be rooted in a self-compassionate approach (26, 29, 30). Conversely, the co-occurrence of chronic pain, depression, and anxiety is a common phenomenon (22, 23), but earlier studies focusing on the relationship between self-compassion and mental health frequently refer to depressive symptoms (7, 8) and rarely address anxiety problems. This study fills this gap through the testing of anxiety as a mental health indicator, which can potentially be reduced due to the self-compassionate approach. Self-kindness, as an embodiment of self-compassion, presents the best reliability of this subscale of the Self-compassion Scale (SCS-PL), both alpha Cronbach's coefficient and omega McDonald's (31), and was at least moderately correlated with other dimensions of this measure. It was dictated by the comfort of the participants because this measure has a six-dimensional structure (self-kindness, self-judgment, common humanity, isolation, mindfulness, over-identification) and is relatively long, consisting of 26 items. Self-kindness is a positive manifestation of a compassionate attitude, contrary to self-judgment as a reflection of a negative approach to yourself, characterized by a critical and condemned attitude toward possessing flaws and shortcomings, as well as being harsh and being intolerant to yourself (32).

We assumed that self-kindness at T1 predicts emotional well-being at T2 as an antecedent of pain intensity and anxiety at T2. Those assumptions were consistent with the broaden-and-build theory of positive emotions (29) and suggestions from previous studies indicating self-compassion as a relevant element of emotional regulation (4, 8, 25, 26), which in turn can predict pain intensity (33, 34) and anxiety (35). Between T1 and T2, an anesthesiologist administered standard pharmacological analgesic treatment without any medication changes, and the patients’ clinical state was controlled. Some of the patients participated in the psychotherapeutic group, which could have a positive impact on some research results, but not on the relationship between them. Other types of non-pharmacological treatment that may influence the outcome were not implemented. Comorbidities were not assessed.

It was hypothesized that self-kindness at Time 1 would positively predict positive affect at Time 2 and would negatively predict negative affect at Time 2 (Hypothesis 1). It was also hypothesized that positive affect at Time 2 would negatively predict pain intensity at Time 2 and anxiety at Time 2, and negative affect at Time 2 would positively predict pain intensity at Time 2 and anxiety at Time 2 (Hypothesis 2). Finally, we expected that self-kindness at Time 1 would be indirectly negatively related to pain intensity and anxiety at Time 2, through positive and negative affect at Time 2 (Hypothesis 3).

## Method

### Participants

The research participants were 58 patients with chronic pain from the Pain Management Clinic of the University Clinical Hospital in Poznań, Poland. The data were collected at two time points: February–May 2025 (T1) and June–July 2025 (T2), with at least a three-month interval. Participants completed the online questionnaire in Google Docs, and some used the pencil-and-paper method as an additional possibility. Participants were eligible if they were aged 18 years or older, had chronic pain understood as persisting for more than 3 months, and a score of 4 or more on the Numerical Rating Scale (NRS) for pain intensity. Patients provided both oral and written informed consent to participate in the study and were informed of the right to withdraw at any time without consequences. The Bioethics Committee of Poznań University of Medical Sciences (approval no. 212/25, dated May 8, 2025) accepted the study protocol. Of the 61 patients who completed questionnaires in the first wave, 58 participated in the second wave. This was due to two withdrawals and one death. The descriptive statistics are presented in Table 1.

**Table 1:** Descriptive statistics for patients with chronic pain (*N* = 58).

Variable	Mean		Standard Deviation		Skewness		Kurtosis		Alpha Cronbach Coefficient		Average Variance Extracted		Composite Reliability	
	T1	T2	T1	T2	T1	T2	T1	T2	T1	T2	T1	T2	T1	T2
Self-kindness	15.00	15.22	4.70	4.39	.01	-.08	-.08	-.04	.81	.76	60.32	57.70	.88	.85
Positive affect	13.36	13.44	3.90	4.23	.17	.39	.62	.13	.68	.81	47.25	57.16	.82	.87
Negative affect	15.91	16.20	5.15	5.05	-.06	-.02	-.97	-.95	.87	.90	65.31	71.94	.90	.93
Anxiety	22.79	21.72	6.56	8.08	-.22	-.10	-.52	-.97	.89	.95	62.05	76.21	.92	.96
Pain severity	5.43	5.48	2.05	2.29	.01	-.14	-.91	-.98	-	-	-	-	-	-

The mean age of participants was 56.7 years (SD = 18.7). The vast majority was Roman Catholic (84.5%). The women outnumbered the men (63.8% versus 36.2%). Primary education had 10.3%, secondary education 46.6%, tertiary education 22.4%, and vocational education 20.7%. The majority of participants were retired (60.3%), 22.4% were employed, 6.9% unemployed, and 10.4% were students. Almost half were married or partnered (46.6%), 15.6% were single, 15.6% widowed, and 22.2% divorced. The distribution of pain localization was following: head (5.2%), Neck (3.4%), thorax (67.2%), lumbar/sacral (67.2%), shoulder (1.7%), upper limb (13.8%), lower limb/hip (24.1%).

### Measures

#### Self-kindness

To assess self-compassion, the Polish version of the Self-compassion Scale (SCS-PL) (31) was used. It has a six-dimensional structure (self-kindness, self-judgment, common humanity, isolation, mindfulness, over-identification) and consists of 26 items, each rated on a 5-point Likert scale (ranging from 1 = “almost never” to 5 = “almost always”). This scale possesses good psychometric properties in terms of internal validity and reliability. In this research, the items regarding self-kindness were applied. The average variance extracted (AVE) for self-kindness items was 60.32% at T1 and 57.70% at T2.

#### Positive and negative affect

Positive and negative affect were examined through the Polish short form (36) of the Positive and Negative Affect Schedule (37). It consists of the five strongest-loading items for positive and negative affect from the original 20-item version of PANAS (38). The answers are provided on a 5-point Likert scale (ranging from 1 = “a little or none” to 5 = “very frequently”). The two-dimensional character of this tool was confirmed, explaining 61.45% of the variance of this construct at T1 and 66.25% at T2. AVE was only slightly below the threshold of 0.5 (39) in reference to positive affect at T1 but above this value regarding positive affect at T2 and negative affect at T1 and T2 (see Table 1).

#### Anxiety

The items from the Polish adaptation (40) of the Generalized Anxiety Disorder Scale (GAD-7) (41) were used to examine anxiety. This measure has a unidimensional 7-item structure. In comparison to the Polish adaptation of GAD-7 and the original version, where responses are rated on a 4-point Likert scale (0—not at all, 1—several days 2—more than half the days, 3—nearly every day), the 5-point Likert scale (1—not at all, 2—several days 3—more than half the days, 4—nearly every day, 5—always) was applied. The difference regarded the additional response “always” and the rating of the response (not within the range from 0 to 3 but from 1 to 5). A response, “always”, was used as an additional possible response as the opposite side of “not at all”, present in the original version of GAD-7, to better cover the full range of responses regarding this measure of anxiety. This means that responses were rated on a 5-point Likert scale (1 = not at all to 5 = always). These response ratings were used in both waves (T1 and T2). The AVE of anxiety regarding T1 and T2 was above 0.5 (see Table 1).

#### Pain intensity

Pain intensity was assessed using Numerical Rating Scale (NRS) items for current pain and average pain over the past week, with 0 indicating no pain and 10 indicating the worst possible pain.

### Statistical analysis and preliminary results

Statistical analysis was conducted using SPSS version 31. Model 4 of the PROCESS macro, authored by Hayes (42, was applied. To assess the direct and indirect effects between study variables, the bootstrap method with 5,000 resamples and a 95% confidence interval (CI) was used (43). According to the theoretical model shown in Figure 1, self-kindness at T1, an independent variable, is indirectly related to anxiety and pain intensity at T2, the dependent variables, via positive and negative affect at T2 as a mediating variable. Within the regression analysis, self-kindness at T1 was the independent variable, anxiety at T2 was the dependent variable; pain intensity and anxiety at T1 were covariates, and positive and negative affect at T2 were mediating variables. Also in the regression model with self-kindness at T1 as the independent variable and pain intensity as the dependent variable at T2, positive and negative affect at T2 were mediating variables, and pain intensity at T1 was a covariate.

**Figure 1:**
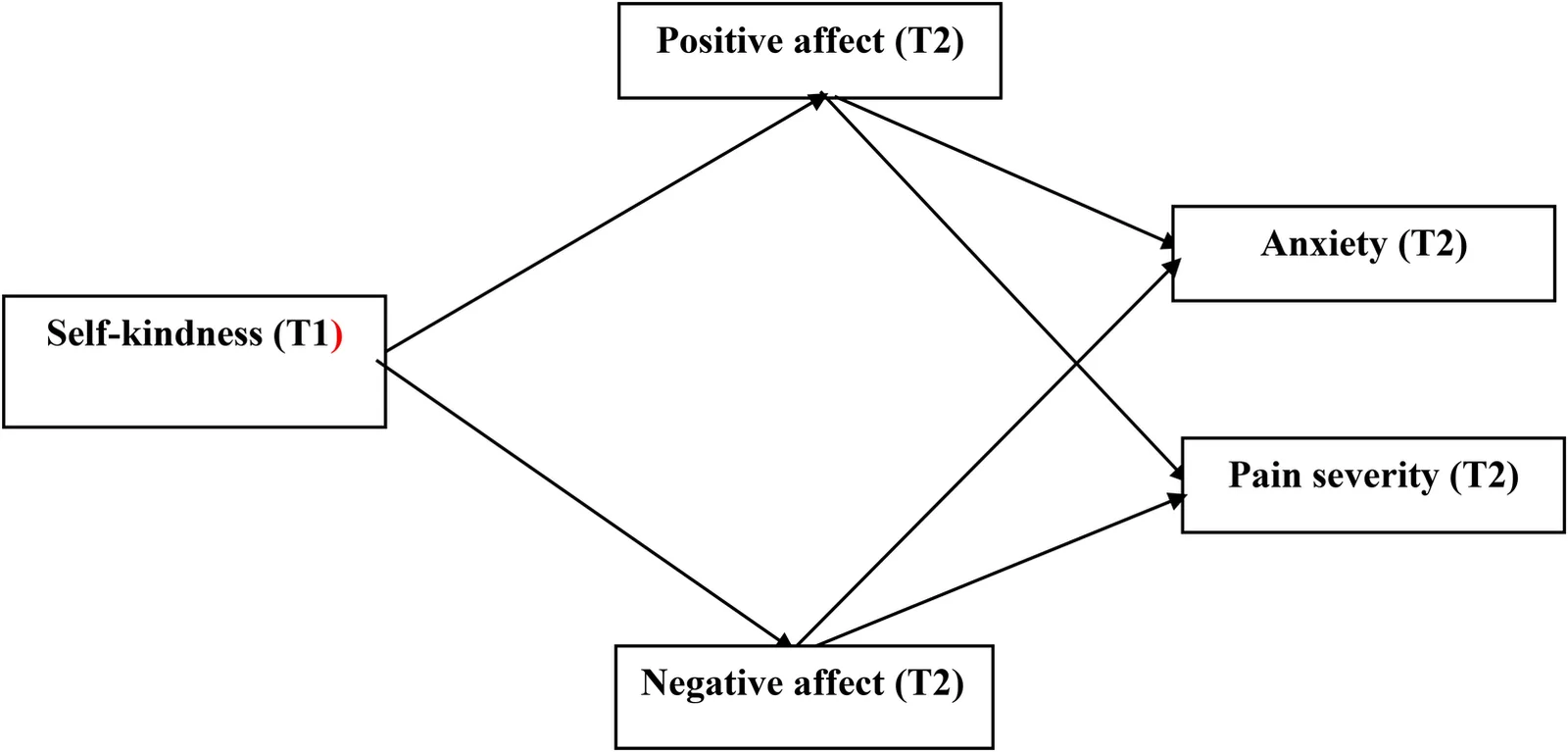
Theoretical model.

The Pearson correlation coefficient is presented in Table 2. Self-kindness at T1 was positively correlated with positive affect at T2, negatively related to negative affect and anxiety at T2, but unrelated to pain intensity at T2. Positive affect at T2 was negatively correlated with anxiety and pain intensity at T2. Negative affect at T2 positively correlated with anxiety and pain intensity at T2.

**Table 2:** Values of r-Pearson correlation coefficients between research variables for patients with chronic pai*n (N* *=* *58)*.

Research variables	1	2	3	4	5	6	7
1. Self-kindness (T1)							
2. Positive affect (T2)	.35**						
3. Negative affect (T2)	-.40**	-.45**					
4. Pain severity (T2)	-.02	-.41**	.26*				
5. Anxiety (T2)	-.31*	-.40**	.81**	.30**			
6. Positive affect—covariate (T1)	.37**	.58**	-.36*	-.11	-.27*		
7. Negative affect—covariate (T1)	-.22	-.17	.56**	.11	.55**	-.32*	
8. Pain severity—covariate (T1)	-.11	-.26*	.13	.54**	.16	-.23	.05

*p *<* .05. **p *<* *.01.*

### Hypotheses testing

The results of the model with self-kindness as the independent variable, positive and negative affect as mediating variables, and anxiety as the dependent variable are presented in Figure 2, but with pain intensity as the dependent variable in Figure 3.

**Figure 2:**
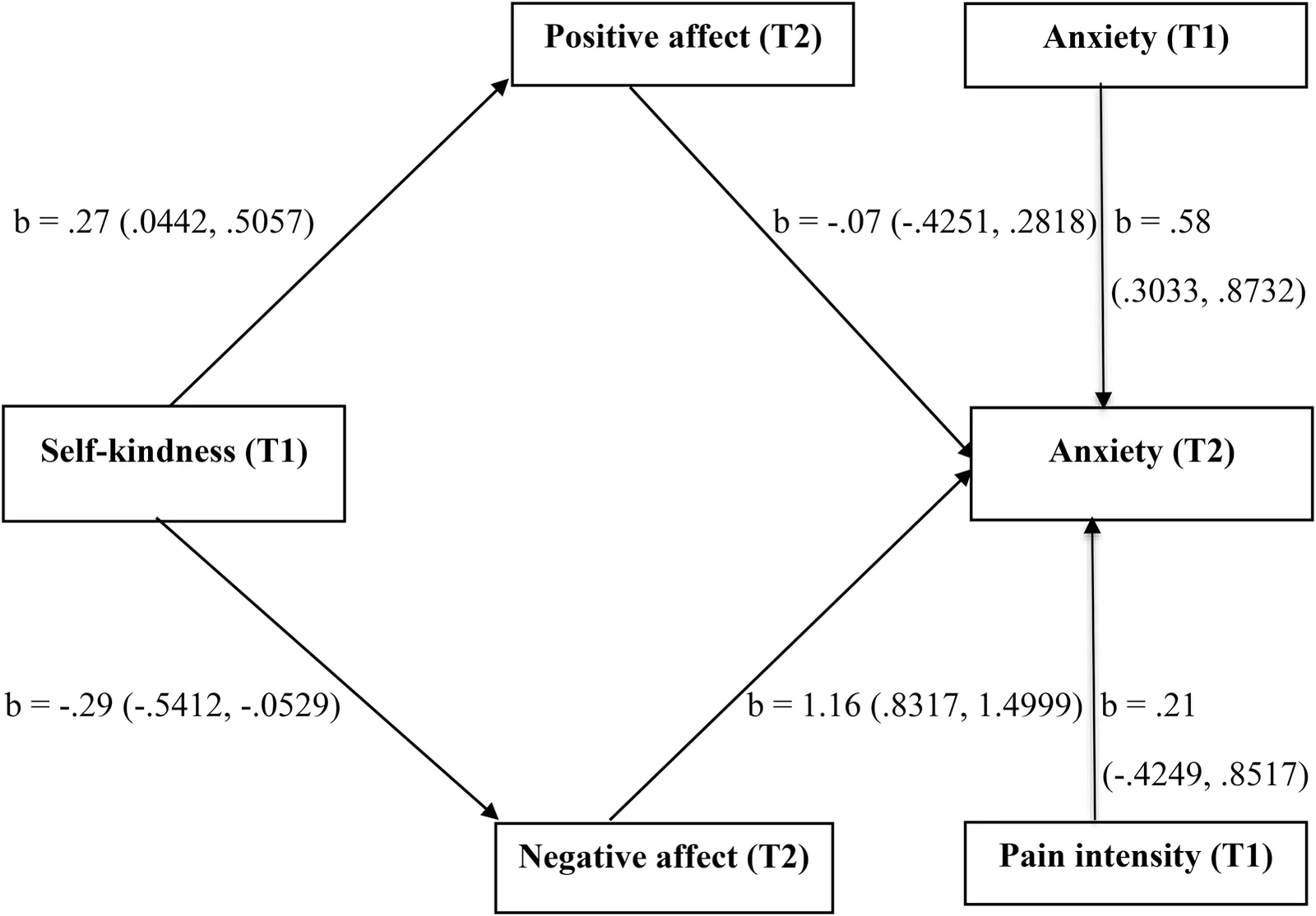
Results of model with self-compassion as independent variable, positive and negative affect as mediating variable, and anxiety as dependent variable. T1 means the times of the first wave study, but T2 means the second wave. b means the unstandardized effect. Anxiety at T1 and Pain intensity at T1 were included as covariates. In parentheses, the values of the lower level of the 95% confidence interval and the upper level of 95% confidence interval are presented. The effect is statistically significant when the 95% confidence interval does not include zero.

**Figure 3:**
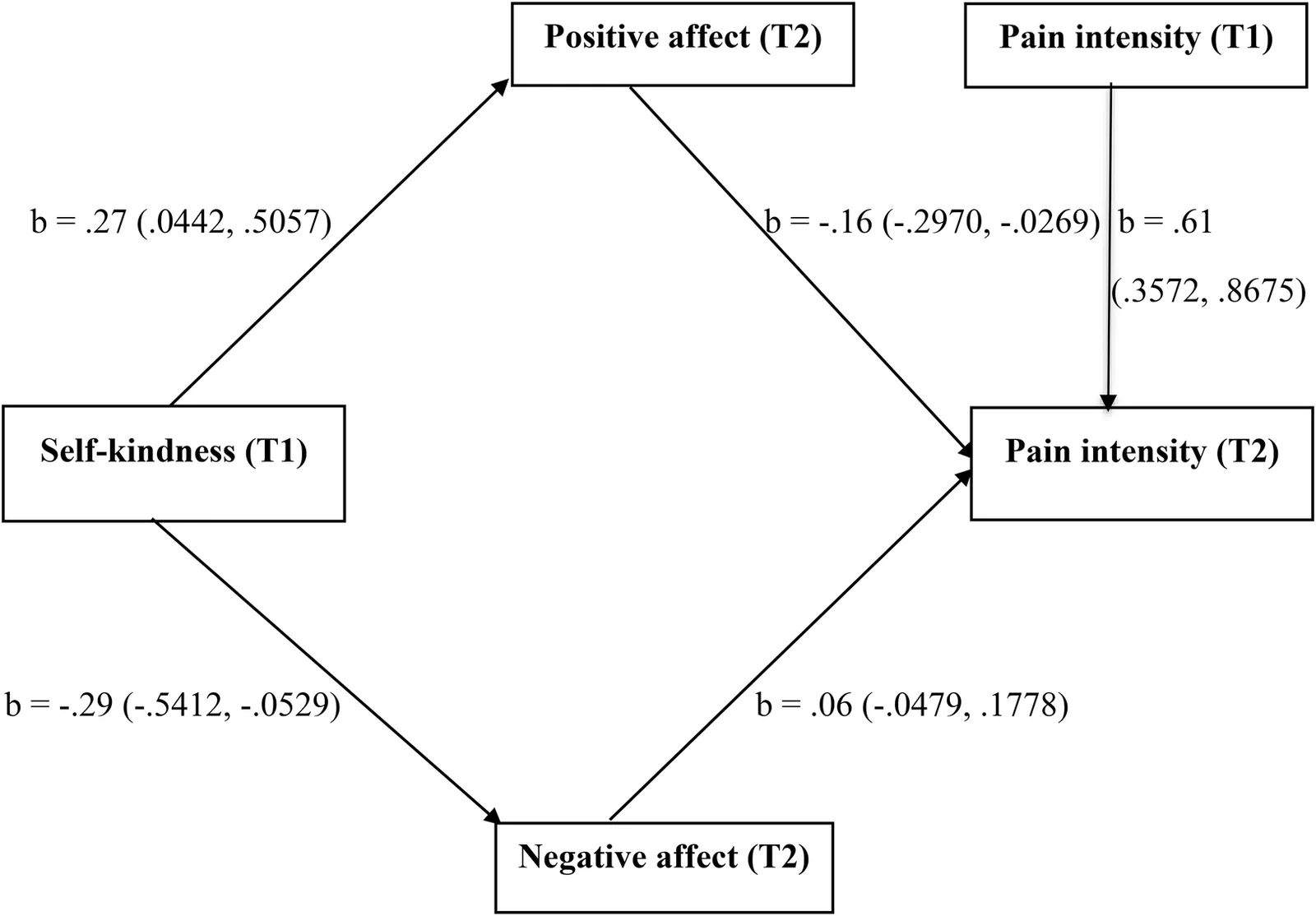
Results of model with self-compassion as independent variable, positive and negative affect as mediating variable, and pain intensity as dependent variable. T1 means the times of the first wave study, but T2 means the second wave. b means the unstandardized effect. Pain intensity at T1 was included as a covariate. In parentheses, the values of the lower level of the 95% confidence interval and the upper level of 95% confidence interval are presented. The effect is statistically significant when the 95% confidence interval does not include zero.

In line with hypothesis 1, self-kidness at T1 positively predicted positive affect at T2 (b = .27, t = 2.38, *p* = 020, CI [.0442,.5057]) and negatively predicted negative affect at T2 (b = -.29, t = -2.43, *p* = 018, CI [-.5412, -.0529]) (see Figures 2, 3). Hypothesis 2, regarding the positive affect at T2 as a negative predictor of anxiety and pain intensity at T2, and negative affect at T2 as a positive predictor of anxiety and pain intensity at T2, was partially confirmed. Positive affect at T2 was not a statistically significant predictor of anxiety at T2 (b = -.07, t = -.40, *p* = 685, CI [-.4251,.2818) (see Figure 2), but statistically significantly predicted pain intensity at T2 (b = -.16, t = -2.40, *p* = 019, CI [-.2970, -.0269) (see Figure 3). Negative affect was found to be a positive predictor of anxiety (b = 1.16, t = 7.00, *p* < .001, CI [.8317, 1.4999) (see Figure 2), but not pain intensity (b = .06, t = 1.15, *p* = 253, CI [-.0479,.1778) (see Figure 3).

Finally, hypothesis 3 that self-kindness at T1 is indirectly related to anxiety at T2 and pain intensity at T2 through positive affect at T2 and negative affect at T2 was partially supported. The results of standardized indirect effects are presented in Table 3. The indirect relationship of self-kindness at T1, through positive affect at T2, on anxiety at T2 was not statistically significant (b = -.01, CI [-.1002,.0336]). The indirect relationship of self-kidness at T1, through positive affect at T2, on pain intensity at T2 was statistically significant (b = -.09, CI [-.2487, -.0027]). The indirect relationship of self-kindness at T1, through negative affect at T2, on anxiety at T2 was statistically significant (b = -.20, CI [-.3676, -.0362]). The indirect relationship of self-kindness at T1, through negative affect, on pain intensity at T2 was not statistically significant (b = -.05, CI [-.1768,.0863]).

**Table 3:** Results of standardized indirect effects for 95% confidence intervals (*N* = 58).

Pathway	Value of effect	LLCI	ULCI
Self-kindness at T1—positive affect at T2—anxiety at T2	-.01	-.1002	.0336
Self -kindness at T1—negative affect at T2—anxiety at T2	-.20	-.3676	.-0326
Self-kindness at T1—positive affect at T2—pain intensity at T2	-.09	-.2487	.-0027
Self -kindness at T1—negative affect at T2 pain intensity at T2	-.05	-.1768	.0863

LLCI, 95% confidence interval (low); ULCI, 95% confidence interval (high).

Direct effect of self-kindness at T1 on anxiety at T2 was not statistically significant (b = .06, t = .40, *p* = 685, CI [-.2398.3621]), the same as the direct effect of self-compassion at T1 on pain intensity at T2 (b = .09, t = 1.63, *p* = 107, CI [-.0211.2091]). The direct effect of pain intensity at T1 as a covariate on anxiety at T2 was not statistically significant (b = .21, t = .67, *p* = 505, CI [-.4249.8517]). Anxiety at T1 as a covariate predicted anxiety positively at T2 (b = .58, t = 4.13, *p* < .001, CI [.3033,.8732) (see Figure 2), the same as the pain intensity at T1 as a covariate predicted pain intensity at T2 (b = .61, t = 4.80, *p* < .001, CI [.3572,.8675) (see Figure 3).

## Discussion

This study was the first attempt to examine the role of positive and negative emotions of patients with chronic pain in the relationships between self-kindness and anxiety, as well as self-kindness and pain intensity. In accordance with previous research, it was found that the self-kindness approach, thanks to better emotional regulation, can be fruitful for patients suffering from persistent pain in terms of anxiety symptoms and pain (17–20).

Importantly, only negative, and not positive emotions, were involved in the indirect relationship between self-kindness and anxiety. On the other hand, self-kindness was indirectly negatively related to pain intensity, through positive, but not negative emotions. It implies that these two pathways differ, suggesting that the beneficial effects of self-kindness may vary in consequences depending on the intensity of positive and negative emotions. It aligns with Park and Sonty’s (44) study, in which pain-related coping efficacy was indirectly related to pain-related interference in social activities through positive affect, but not negative affect. Also, they were consistent with previous studies indicating that positive emotions are negatively related to pain in patients with chronic pain (45–47). Additionally, they are partially coherent with earlier studies, showing that negative affectivity is a stronger predictor of anxiety symptoms than positive affectivity (35). The lack of a significant association between positive affect and anxiety was probably caused by the simultaneous presence of negative affect in the regression analysis, because in pairwise correlation (see Table 2), positive affect correlated moderately and negatively with anxiety.

An appropriate interpretative framework for this study's outcomes is the broaden-and-build theory of positive emotions (29). In line with this theoretical approach, self-kindness is associated with reduced negative affect and elevated positive affect, suggesting that, in accordance with recent research, self-kindness may serve as an effective coping method and facilitator of emotion regulation (48, 49). The self-kindness approach may be favorable for positive emotions by treating painful feelings with kindness and aiding the transformation of emotional suffering into a manageable experience. This aim can also be achieved by providing a safe emotional space that motivates, rather than pressures, improvement (50). Finally, a self-kindness attitude can limit negative emotions by mitigating self-criticism and rumination, thereby weakening emotional reactivity, which is accelerated by the cycle of shame and guilt, and can break social isolation by understanding that suffering is an inherent element of human existence.

The main theoretical contribution of this study is to confirm the indirect relationships between self-kindness and anxiety, as well as self-kindness and pain intensity in patients with chronic pain. It suggests that a self-kindness approach is associated with increased positive emotions, which, in turn, may negatively predict pain intensity. Also, self-kindness may be indirectly related to anxiety, via negative emotions. Previous studies have emphasized the benefits of self-compassion for pain experience and mental health in this group of patients, but without presenting the indirect effect through negative emotions. It needs to be emphasised that despite using the bootstrapping method in this research, it should be interpreted with caution because of the small sample size and single-center recruitment, which probably weakened the statistical power and their generalizability. Additionally, heterogeneous chronic pain diagnoses among the study sample included in the study would also provide variability, what make a difficulties and in applicability of the findings to specific chronic pain patient populations. This means that the generalizability of this study could not have been so limited even in a small sample, when it was homogeneous in terms of chronic pain diagnoses.

The practical contributions of this research indicated self-kindness as a facilitator of emotional regulation, which promote decreas of negative affect and an increase in positive affect in chronic pain. It can be considered by psychologists, psychotherapists, anesthesiologists, social workers, and counselors to implement therapeutic interventions aimed at enhancing the self-kindness of their patients. They can be provided both in group therapy and in individual visits as potential support for pharmacological treatment. Some techniques focused on improving self-compassion are relatively easy to use and do not require specialized therapeutic skills (21), making them available for patients to practice. It means that specialists caring for patients with chronic pain, and their support systems for managing pain, can also motivate patients to develop this strength of character on their own. From the clinical practice point of view, a suitable approach to patients with chronic pain focused on building the patients’ image as someone who experiences suffering and requires relief and self-caring could be an effective coping strategy with negative emotions, such as guilt, remorse, or fear. It seems especially important in patients who blame themselves for their illness as a source of pain, and ruminate and reconsider mistakes they made in the past instead of searching for hope and ways to deal with their pain, as well as building a compassionate attitude toward themselves. It is also essential to examine whether self-kindness intervention will lead to less pain intensity and anxiety symptoms, not only improving emotional well-being. This study shows only indirect effects through positive and negative affect. Future research should explore the relationship between self-compassion and pain as well as mental health in an experimental way.

The second area of potential improvement regards emotional regulation as accompanied by pain and anxiety experienced by patients with chronic pain. Due to experience positive emotions, patients may experience physical pain as less intense and burdensome. It suggests implementing training focused on inducing positive emotions, as a possible method in struggling with pain, but it requires further exploration (46). On the other hand, within professional support, patients may learn how to deal with negative emotions to cope with anxiety, which frequently co-occurs with persistent pain (22, 23).

### Limitations and future research

The first limitation concerns the generalizability of the research outcomes. The participants were patients at one clinic for chronic pain treatment with various diagnoses. Additionally, the sample was small, suggesting that future research should use a more representative sample of chronic patients with homogeneous medical diagnoses. Also, religious socialization, prevalent in Poland, could affect the study outcomes, especially regarding self-kindness, which may be approached differently among individuals without religious affiliation or even across religious representatives. It requires conducting future research in a more secular and more heterogeneous religious background.

Since this study uses items from only one self-compassion dimension, it is necessary to employ the others, which may be in other ways related to affectivity, anxiety, and pain. To confirm the examining mechanisms from the cause and the effective perspectives, the third wave of research should be implemented in which the anxiety symptoms and pain intensity should be measured at T3, not only at T2. The absence of verification of the level of anxiety and pain intensity at T3 indicates an indirect relationship of self-kindness through emotional wellbeing, but not a mediating effect, even though self-kindness was verified in T1 and positive and negative affect at T2. It means that the obtained results do not confirm whether increasing positive affect leads to less pain intensity and reducing negative affect causes less anxiety, but indicate the coexistence of those phenomena. Additionally, the inverse direction between self-kindness and affectivity, and affectivity and anxiety and pain, was not considered. It is possible that affectivity influences self-kindness. Moreover, pain intensity, as well as anxiety, may lead to less positive affect and more negative affect. Moreover, other indirect pathways were not considered in the study, such as how emotional well-being can be indirectly related to self-kindness via anxiety and pain, or how self-kindness can be negatively related to pain intensity and anxiety, which in turn may predict emotional well-being. Only the future three-wave study would identify the full mediation effect, not only the indirect effect and show causal direction between these variables.

Future research needs to verify this claim. Also, other pain-related indicators besides physical pain should be considered, such as pain acceptance, pain interference, or pain catastrophizing (20). Finally, positive mental health indicators should be used to examine whether self-compassion is indirectly related to well-being through elevated positive affect, inhibited negative affect, or both.

## Data Availability

The raw data supporting the conclusions of this article will be made available by the authors, without undue reservation.
